# Primary leptomeningeal histiocytic sarcoma in a patient with a good outcome: a case report and review of the literature

**DOI:** 10.1186/1752-1947-7-127

**Published:** 2013-05-13

**Authors:** Elisabeth Pérez-Ruiz, Maite Delgado, Andrés Sanz, Ana Maria Serradilla Gil, Antonio Rueda Domínguez

**Affiliations:** 1 Division of Medical Oncology, REDISSEC, Hospital Costa del Sol, Autovía A-7, Km 187, Marbella, C.P. 29603, Spain; 2 Pathology Department, Hospital Universitario Carlos Haya, Málaga, Spain; 3 Division of Radiotherapy Oncology, Clinic Croasa, Málaga, Spain

**Keywords:** Chemotherapy, Histiocytic sarcoma, Leptomeningeal sarcomas, Multidisciplinary treatment, Radiotherapy

## Abstract

**Introduction:**

Histiocytic sarcoma is a rare neoplasm with few cases reported in the literature of which some were diagnosed in animals. This neoplasm arises from abnormal reticuloendothelial system cell proliferation of histiocytes and has an aggressive behavior especially if located in the central nervous system. We present the first case of a patient with histiocytic sarcoma that involved the meninges and had a good course after multidisciplinary treatment.

**Case presentation:**

Our patient was a 41-year-old Caucasian woman with no previous history of disease who started with systemic symptoms such as headache and chills. Magnetic resonance imaging with gadolinium contrast of the brain suggested a mass 1.5×2cm in diameter in the temporal lobe with a non-uniform vasogenic edema. This lesion was implanted in the meninges and surgery was the first treatment. The histological findings revealed a histiocytic sarcoma. The patient received concomitant chemoradiotherapy after surgery with good tolerance and currently lives without disease.

**Conclusion:**

Although histiocytic sarcomas in the brain present an unusual location and have a poorer prognosis, we have identified the first primary leptomeningeal histiocytic sarcoma with a disease-free survival greater than 3 years following multidisciplinary treatment with surgery and chemotherapy and radiotherapy.

## Introduction

Histiocytic sarcoma (HS) is a rare neoplasm showing evidence of histiocytic differentiation. It often appears in the skin, lymph nodes, and intestinal tract but central nervous system (CNS) involvement is rare. The standard treatment for these sarcomas is surgery. The best option for treating these tumors is unclear and affected patients have a poor prognosis despite treatment.

## Case presentation

**Figure 1:**
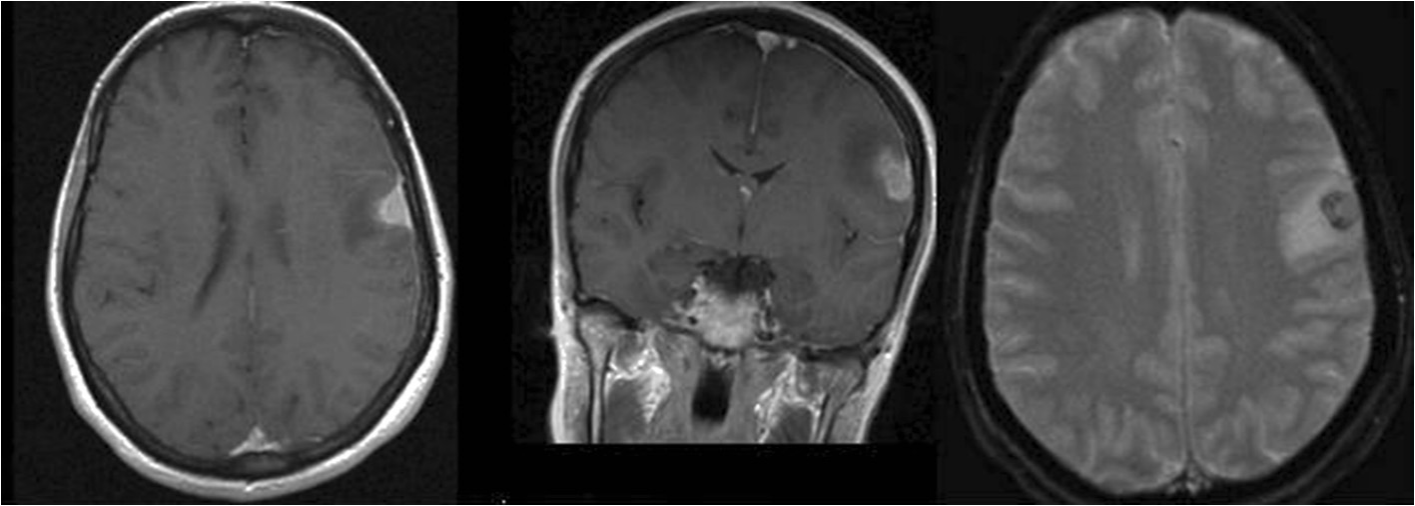
**Magnetic resonance imaging demonstrating meningeal involvement and possible neoplastic disease.** From left to right: Axial T1 image, coronal T1 image, and axial T2 image.

**Figure 2:**
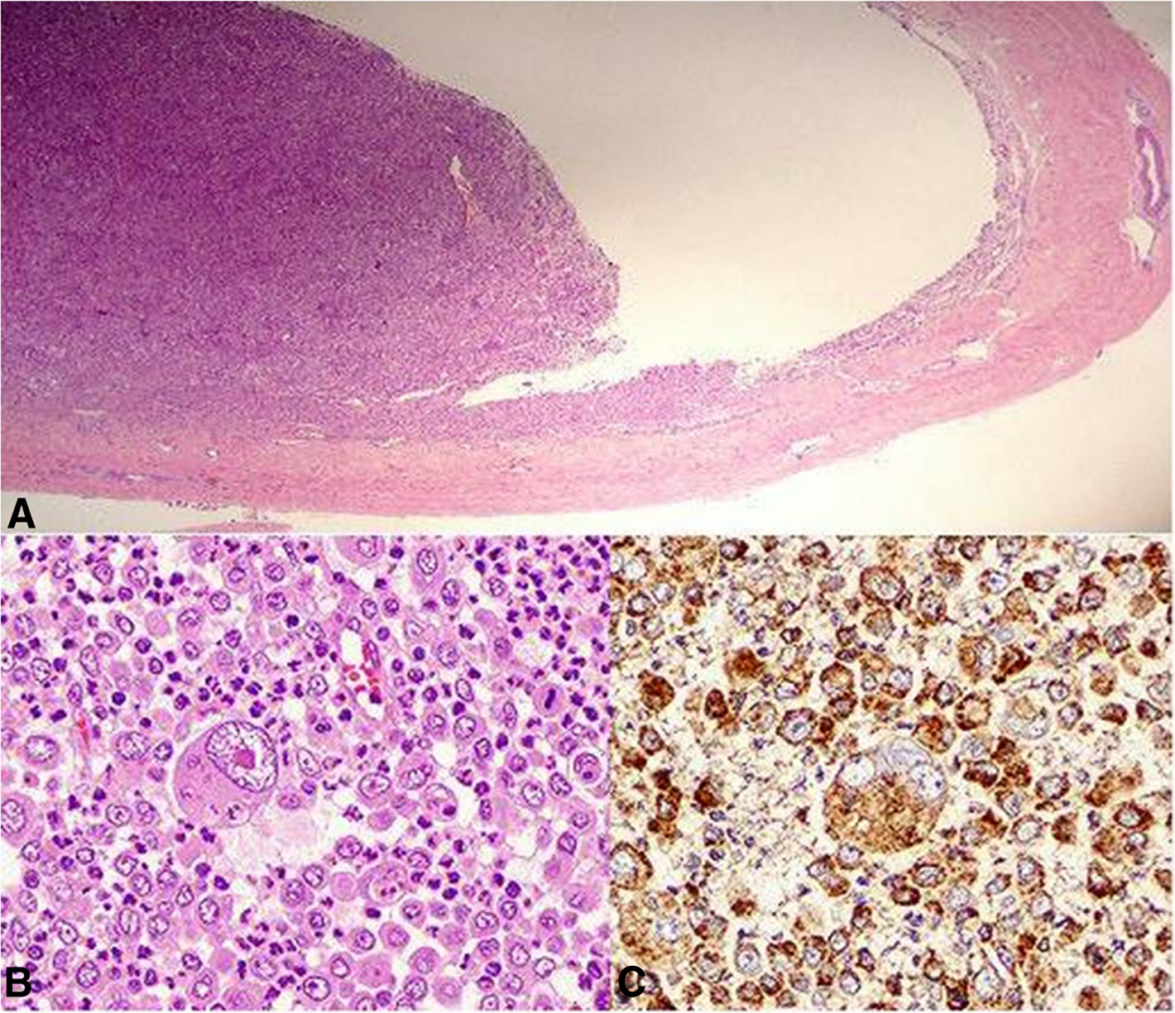
** Histologícal findings. A**. Laminar fragment corresponding to dura mater (right) on which arises the tumor. **B**. Diffuse non-cohesive proliferation of neoplastic cells that look like a histiocyte. The cells are variable in size, with irregular nuclei and a strikingly large central nucleus. There is a lymphocyte and neutrophil inflammatory background showing numerous neutrophils and phagocytosis by tumor cells. **C**. Positive expression of CD68 in tumor cells.

In June 2009, a 41-year-old Caucasian woman with no previous history of illness went to the Emergency Room with systemic symptoms, including headache, generalized weakness, and chills. The physical examination showed nothing remarkable. The workup included a computed tomography (CT) brain scan without contrast that showed nothing unusual. The patient received empirical treatment for a diagnosis of a suspected viral infection. A week later, she presented with dysphasia and deviation of the right corner of her mouth. Magnetic resonance imaging (MRI) with gadolinium contrast of the brain suggested a mass 1.5×2cm in diameter in the temporal lobe with a non-uniform vasogenic edema. This lesion was implanted in the meninges and indicated possible meningioma (Figure 1). There was no evidence of malignancy on chest, abdominal or pelvic CT. In July, left frontal craniotomy surgery was performed. It showed a meningeal lesion in the dura mater extending to nearby tissue. The lesion was excised in its entirety (Figure 2A). The histological finding described a lesion in the meninges with a diameter of 3×2cm. The histological findings also revealed a diffuse non-cohesive proliferation of neoplastic cells that looked like a histiocyte. The cells were variable in size, with large and abundant foamy eosinophilic cytoplasm. The cytoplasm presented numerous neutrophils and phagocytosis by tumor cells. The nuclei were irregular and large with mono- or multi-nucleation displaying a vacuole appearance with granular chromatin. It frequently presented prominent eosinophilic nucleoli. There was a lymphocyte and neutrophil inflammatory background (Figure 2B). The entire lesion was neoplastic. The pathologist suggested a lymphoma but an immunohistochemistry study was done the results of which showed positive expression of CD68 (Figure 2C), CD163 and lysozyme consistent with histiocytic lineage and weak expression of S100. The cells, however, presented negative expression of cytokeratin, B or T cells, and myeloid markers, including anaplastic lymphoma kinase 1 (ALK-1), CD3, CD4, CD8, CD20, CD21, CD23, CD30, CD1a, Bcl2, Bcl6, CD15, CD31 and CD10. The polymerase chain reaction study did not show clonal B or T cells. After these results, the pathologist diagnosed a HS. We presented the case in the multidisciplinary meeting and we decided on adjuvant treatment with chemoradiotherapy: temozolomide (120mg daily) concomitant with 1.8Gy of radiotherapy 5 days a week. The radiotherapy was intensity-modulated radiation therapy guided by imaging: planning target volume (PTV)1 received 45Gy, PTV2 received 54Gy and PTV3 61.2Gy. The patient remains disease free after 42 months.

## Discussion

HS also formerly known as “true” histiocytic lymphoma is a rare neoplasm showing evidence of histiocytic differentiation. It often appears in the skin, lymph nodes, and intestinal tract [1]. Although CNS involvement is rare, there are a few cases reported in the literature [1-5] and other cases diagnosed in animals, such as cats, dogs, and camels [6-9]. The cases involving the CNS occurred by continuation of the disease except the case reported by Torres *et al*. [5] which appeared in the subarachnoid space.

HS has often been classified as being similar to diseases such as malignant histiocytosis, histiocytic lymphoma, and reticular sarcoma, based on morphological criteria [10]. However, advances in molecular and biological markers have improved immunohistochemistry findings, allowing for differentiation between HS and B or T cells and non-Hodgkin lymphomas. These diseases have a prognosis and treatment different from sarcomas.

HS is composed of mononuclear tumor cells with large cytoplasmic vacuoles. Some cells present with phagocytosis phenomena. The diagnosis is based on immunohistochemistry markers of histiocytic cells such as positive CD68, lysozyme, α1-antitrypsin and CD163, which is regarded as a specific HS marker [11]. However, HS presents negative markers of epithelial (epithelial membrane antigen or cytokeratin), melanocytic (CD34 or myeloperoxidase) and lymphoid (CD3 or CD20) neoplasms as in our case. Markers of Langerhans cell (CD1a), follicular dendritic cells, B cells, T cells, and myeloid cells are negative. Lack of expression of CD30 and ALK-1 excludes anaplastic large cell lymphoma, which is an important differential diagnosis. The focal staining for S-100 protein is not uncommonly seen in the true histiocytic lymphomas as reported in the literature [12].

Radiological findings have not been established due to the low number of cases. The HS image study reported in the literature addressing the brain is MRI [7], which describes the extent of the disease. The images point towards neoplastic disease, but there are no specific images suggesting HS. Some MRI studies in dogs are inconclusive because the image presented could be either a benign process (such as encephalitis) or a malignant process (such as meningioma) [6].

The standard treatment for these sarcomas is surgery. The best option for treating these tumors is unclear. Furthermore, when the tumor is located in the CNS the therapeutic options are reduced. Brain sarcomas have an aggressive course with an overall survival rate of around 4 to 5 months, regardless of treatment. There are no studies where the benefit of radiotherapy or chemotherapy for HS has been established.

In our case, the patient received concomitant chemoradiotherapy after surgery. We decided to administer temozolomide with the radiotherapy because the patient was young and she presented with a sarcoma affecting the CNS. It is also known that this type of chemotherapy crosses the blood–brain barrier and it has shown benefit over radiotherapy alone in other aggressive tumors [13]. She had a good tolerance and currently lives without disease (disease-free interval of 42 months). The cases reported before were treated with different schedules of chemotherapy by the vein or with intrathecal treatment but none received concomitant radiotherapy. All the cases had an aggressive course except one case that relapsed after 3 years [1]. In the literature, some cases of extranodal HS treated with surgery and some type of chemotherapy or radiotherapy had a good outcome. These reported patients lived varying months after treatment [14]. Recently, a radiotherapy-induced HS has been treated with radiation alone and the patient remained free of recurrent disease after 1.5 years [15]. Radiation alone could be beneficial but we do not know whether radiation-induced HS differ in their biological behavior.

## Conclusion

In conclusion, HS is a rare neoplasm with a diagnosis based on immunohistochemistry and biological findings. Although HS in the brain present an unusual location and have a poorer prognosis, we have identified the first primary leptomeningeal HS with a disease-free survival greater than 3 years following treatment with surgery and chemotherapy and radiotherapy.

## Consent

Written informed consent was obtained from the patient for publication of this case report and accompanying images. A copy of the written consent is available for review by the Editor-in-Chief of this journal.

## Competing interests

The authors declare that they have no competing interests.

## Authors’ contributions

EPR and AR analyzed and interpreted the patient data regarding the sarcoma histiocytic disease and the treatment. AS performed the histological examination of the brain, and was a major contributor in writing the manuscript. MD and AMS treated the patient. All authors read and approved the final manuscript.
